# Synthesis and Characterization of Boron Nitride Thin Films Deposited by High-Power Impulse Reactive Magnetron Sputtering

**DOI:** 10.3390/molecules29225247

**Published:** 2024-11-06

**Authors:** Vytautas Stankus, Andrius Vasiliauskas, Asta Guobienė, Mindaugas Andrulevičius, Šarūnas Meškinis

**Affiliations:** Institute of Materials Science, Kaunas University of Technology, K. Baršausko St. 59, LT-51423 Kaunas, Lithuania; andrius.vasiliauskas@ktu.lt (A.V.); asta.guobiene@ktu.lt (A.G.); mindaugas.andrulevicius@ktu.lt (M.A.)

**Keywords:** hexagonal boron nitride, reactive high-power magnetron sputtering, Raman, X-ray photoelectron spectroscopy, AFM

## Abstract

In the present research, hexagonal boron nitride (h-BN) films were deposited by reactive high-power impulse magnetron sputtering (HiPIMS) of the pure boron target. Nitrogen was used as both a sputtering gas and a reactive gas. It was shown that, using only nitrogen gas, hexagonal-boron-phase thin films were synthesized successfully. The deposition temperature, time, and nitrogen gas flow effects were studied. It was found that an increase in deposition temperature resulted in hydrogen desorption, less intensive hydrogen-bond-related luminescence features in the Raman spectra of the films, and increased h-BN crystallite size. Increases in deposition time affect crystallites, which form larger conglomerates, with size decreases. The conglomerates’ size and surface roughness increase with increases in both time and temperature. An increase in the nitrogen flow was beneficial for a significant reduction in the carbon amount in the h-BN films and the appearance of the h-BN-related features in the lateral force microscopy images.

## 1. Introduction

Two-dimensional (2D) nanomaterials, such as graphene, boron nitride (BN), and molybdenum disulfide (MoS_2_) nanosheets, have many unique properties that can be useful for various applications, such as composites, nanoelectromechanical systems, and sensing, optoelectronic, and electronic applications. BN can form several different allotropes with either sp^2^ or sp^3^ bonding. The sp^2^-bonded BN crystallizes in a hexagonal (h-BN) or rhombohedral (r-BN) phase, and sp^3^ BN crystallizes in a cubic (c-BN) or wurtzite (w-BN) phase [1]. Hexagonal boron nitride (h-BN) is a layered 2D nanomaterial that is structurally analogous to graphene [2]. It has excellent physical properties, such as an ultra-wide bandgap (~5.96 eV) [3], a high breakdown field (11.8 MV cm^−1^) [4], high thermal conductivity (1000 W m^−1^K^−1^) [5], good thermal and chemical stability [6], and piezoelectricity [7]. BN nanostructures also present excellent mechanical properties [8]. The atomically thin layer can be assembled with various other 2D layers to create tunneling-based devices, vertical or in-plane heterostructures, and bistable memory devices [9,10,11]. In particular, graphene and h-BN share very similar hexagonal crystal lattice parameters, enabling epitaxial growth of low-defect-density graphene on boron nitride [12]. Therefore, graphene/h-BN heterojunctions and multilayer-based microelectronic and photonic devices are intensively studied [9]. In addition, hBN itself is a promising material for such applications as ultraviolet-light emitters [13], single-photon emitters [14], gas barrier films [14], and tunnel magnetic resistance devices [15]. Boron nitride thin films are synthesized using various deposition techniques. Chemical vapor deposition is the most widely used method for the large-scale production of h-BN layers at a low cost [16,17,18]. However, this technique uses transition metals (Cu, Ni, Fe, Pt, and Ir) as substrates, and a transfer process from metal substrates to a suitable surface (generally a dielectric substrate) is required for most device applications, which probably induces impurities and mechanical damage, thereby degrading the performance of h-BN-based devices. The h-BN films deposited by metalorganic vapor-phase epitaxy [19,20,21] and molecular beam epitaxy [22,23] are usually grown on sapphire, which requires high substrate temperatures (>1000 °C) to compensate for this substrate’s poor catalytic activity. Furthermore, h-BN has been synthesized at lower temperatures by applying physical vapor deposition methods such as radio frequency (RF) magnetron sputtering [24]. However, RF magnetron sputtering is known for low power efficiency, high cost, and constraints in terms of scaling to large surface areas [25]. Thus, the development of other physical-vapor-deposition-based hexagonal boron nitride deposition methods is necessary.

Notably, high-power impulse magnetron sputtering (HIPIMS) is already successfully used for large-area industrial-scale coating deposition [26]. Compared to RF magnetron sputtering, high-power impulse magnetron sputtering ensures higher thin-film density [27,28], much better control of the structure and stoichiometry [29], enhanced adhesion [28], and higher stability [30]. However, there are few studies on h-BN film deposition by HIPIMS [31,32,33,34,35,36]. In addition, in [31,32,34], LaB_6_ targets were used for h-BN growth, because the pure boron target is insulating and, usually, it should be heated to a temperature of 500 °C or higher to ignite the unipolar sputtering discharge [31,32,34,37]. In the case of hexagonal boron nitride deposition by boron target reactive HIPIMS, only Ar and N_2_ gas flow ratio effects [36] and the influence of the boron isotope used as a sputtering target material [33] were studied.

The aim and objectives of this research work are to investigate the synthesis of h-BN thin films directly on noncatalytic Si(100) substrates by applying the reactive unipolar high-power impulse magnetron sputtering (HiPIMS) technique, using a pure boron cathode and nitrogen gas, and to investigate the influence of the deposition temperature and time and the flow rate of nitrogen gas on the structure and composition of thin films. It was revealed that there is no need for high-temperature heating of the boron target to ignite the sputtering discharge, and the target temperature of 100 °C is enough for that purpose. Taking into account the finding in [36] that h-BN can be grown using nitrogen gas alone instead of the Ar/N_2_ gas mixture and the fact that the use of the Ar/N_2_ gas mixture provides no benefits, in the present research, boron nitride films were deposited using N_2_ as both reactive and sputtering gas. However, it was revealed that the nitrogen gas flow must be maximized to minimize the concentrations of unwanted impurities, such as carbon and oxygen, in the film. It was found that the h-BN films’ deposition temperature and time also influenced the h-BN structure and composition. Control of the h-BN nanocrystallite size and decreased intensity of the samples’ Raman spectra luminescence hump and background were achieved.

## 2. Results and Discussion

**Figure 1:**
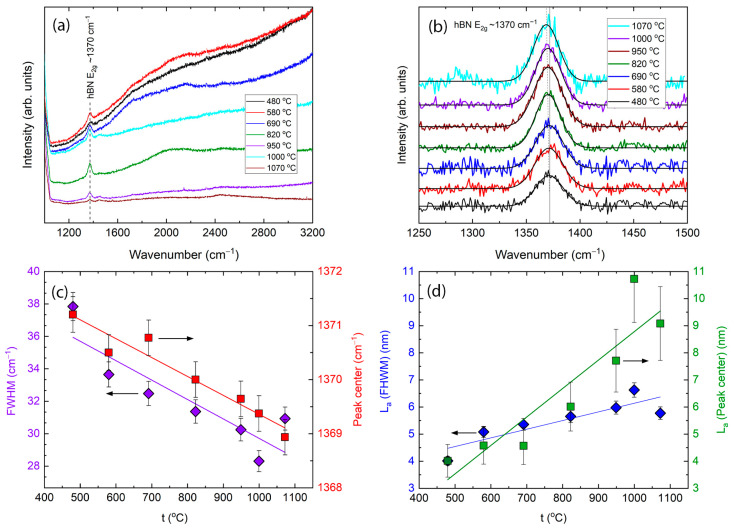
(**a**) Raman spectra of the h-BN films grown at different substrate temperatures and 60 min deposition time. (**b**) Raman spectra of the main peak with removed background and deconvoluted curves. (**c**) FWHM and peak center position dependence on deposition temperature. Crystallite sizes calculated using FWHM and central peak position shifting (**d**).

In the present research, the effects of the deposition temperature and time and the nitrogen gas flow on the structure of h-BN films were investigated by Raman scattering spectroscopy. Figure 1a shows the original Raman spectra of the deposited h-BN films grown at different substrate temperatures, with a 60 min deposition time and a 152 sccm nitrogen gas flow. 

The main peak at ~1370 cm^−1^ is observed at all deposition temperatures. It can be assigned as an h-BN E2g peak related to in-plane, Raman-active vibrations [38,39]. No h-BN-related peaks were observed in the spectra of the samples grown at temperatures below 480 °C. In spectra of the films grown at lower temperatures, a strong luminescence hump and luminescence background are seen, which decreases and almost disappears at higher substrate temperatures. Figure 1b shows a view of the hexagonal boron nitride-related peaks with a removed background and deconvoluted curves. The peak’s central position is down-shifted with the deposition temperature. Fitted values of full width at half maximum (FWHM) and the peak’s central position are shown in Figure 1c. FWHM decreases from 38.01 ± 0.96 to 28.13 ± 0.96 cm^−1^ wavenumbers with increasing temperature, while central position, as was mentioned above, shifts from 1371.3 ± 0.3 to 1368.8 ± 0.3 cm^−1^ wavenumber. Figure 1d shows crystal sizes calculated using Equations (1) and (2). The size of the crystallites calculated using FWHM increases from 4.50 ± 0.46 to 6.50 ± 0.46 nm with increasing deposition temperature from 480 to 1070 °C. A similar effect of crystallite size depending on substrate temperature was reported for h-BN films deposited by RF sputtering [40]. Crystallite sizes calculated using h-BN peak position rise with synthesis temperature from 4.50 ± 1.21 to ~10.00 ± 1.21 nm. We suppose that peak position shifting is affected by two factors—change in the crystallite sizes [41,42,43] and strains induced in the film [44,45]. So, the larger size of the crystallites calculated using the h-BN peak position can be explained by strain appearance in the films grown at higher temperatures, which results in additional peak shifts. There are theoretical studies about the grain size and stress relationship in BN which have established that strength, toughness, Young’s Modulus, and energy release rate all have a declining trend along with a decrease in grain size. At the same time, the ultimate strain increases as grain sizes decrease. These properties stem from the heterogeneity of BN, and the effect of this heterogeneity on the behavior of grain boundaries [46] and tensile strength and strain decreased after introducing vacancy defects in the hBNNR structure [47]. In our case, the grain boundaries play the same role as vacancies—the larger the crystallites, the less grain boundaries between them.

Figure 1 shows that larger crystallite values were calculated using the peak position compared to those obtained using the peak FWHM for samples deposited at temperatures higher than 900 °C. Boron nitride tensile strain results in E2g Raman peak downshift [38] and compressive strain—in upshift [48]. Thus, the presence of the tensile strain in boron nitride samples grown at temperatures higher than 900 °C can be supposed. That can be explained by the thermal stress appearance during the cooling due to the different thermal expansion coefficients of boron nitride and silicon [49,50]. Therefore, to avoid possible adverse effects of excessive thermal stress while maximizing h-BN crystallite size, the boron nitride growth temperature was set at 820 °C in subsequent experiments. Deposition time and nitrogen gas flow effects were investigated. 

**Figure 2:**
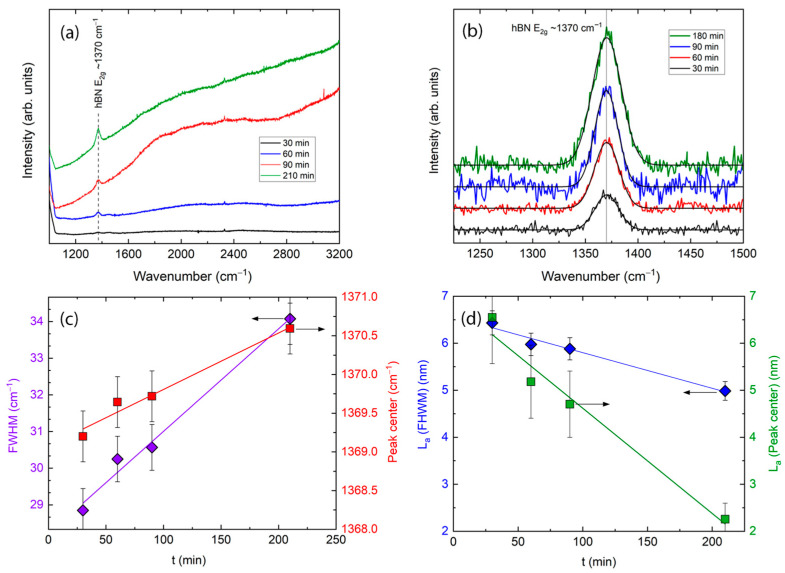
(**a**) Raman spectra of the h-BN films grown at different deposition times at constant substrate temperature 820 °C. (**b**) Raman spectra of the main peak with removed background and deconvoluted curves. (**c**) The dependence of the FWHM and peak center position on deposition time. Crystallite sizes (calculated using FWHM and central peak position shifting) (**d**).

Figure 2a shows the original Raman spectra of the deposited h-BN films, grown at different times (from 30 to 180 min), 820 °C deposition temperature, and 152 sccm nitrogen gas flow. We see the main peak of hBN, attributed to ~1370 cm^−1^ of wavenumber. We can see the increase in luminescence hump with increasing deposition time. Figure 2b shows the high resolution of main peaks with removed background and deconvoluted curves. The broadened and slightly shifted peaks can be seen; we also see an increase in intensity with increasing deposition time. Fitted values of FWHM and the peak’s central position are shown in Figure 2c, which shows that FWHM increases from 28.80 ± 0.96 to 34.50 ± 0.96 cm^−1^ of wavenumber with increasing deposition time, while central position, as was mentioned above, shifts from 1371.2 ± 0.3 to 1368.9 ± 0.3 cm^−1^ wavenumber. Figure 2d shows calculated crystal sizes. Using the calculation from FWHM, the crystallites’ size decreases from 6.5 ± 0.46 to 4.8 ± 0.46 nm with increasing deposition time from 30 to 180 min. Using calculations from peak center shifting, we see that crystallite sizes change from 5.5 ± 0.96 to 2.3 ± 0.96 nm, and the inclination of dependence is different. As was described above, the different inclinations can be explained by the strain effect appearing in crystallites.

**Figure 3:**
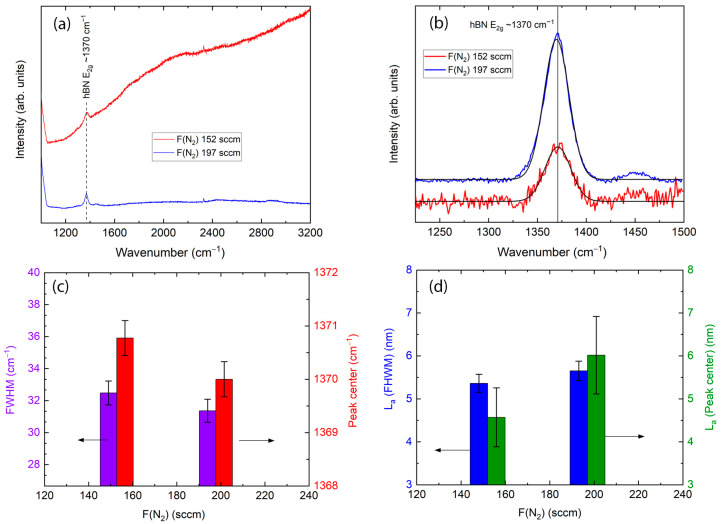
(**a**) Raman spectra of the h-BN films grown at different nitrogen gas flows at constant substrate temperature 820 °C and deposition time 60 min. (**b**) Raman spectra of the main peaks with removed background and deconvoluted curves. (**c**) FWHM and peak center position dependence on gas flow. Crystallite sizes calculated using FWHM and central peak position shifting (**d**).

Figure 3a shows the original Raman spectra of the deposited h-BN films, grown using different nitrogen gas flows at a constant 820 °C deposition temperature and deposition time of 60 min. We see the main peak of hBN, attributed to ~1370 cm^−1^ of wavenumber. A luminescence hump is clearly visible in the spectra of the sample deposited with 152 sccm of nitrogen gas flow. Increasing flow to 197 sccm gives the disappearance of the luminescence hump and background. Figure 3b shows the high resolution of main peaks with removed background and deconvoluted curves. An increase in intensity with increasing nitrogen gas flow is observed. Fitted values of FWHM and the peak’s central position are presented in Figure 3c, which shows that FWHM decreases from 32.50 ± 0.96 to 31.40 ± 0.96 cm^−1^ with increasing gas flow. The h-BN peak position is slightly downshifting. Figure 3d shows calculated crystal sizes. With increasing nitrogen gas flow, the crystallite size calculated using FWHM values increases from 5.36 ± 0.46 to 5.65 ± 0.46 nm. The crystallite sizes estimated using h-BN peak position raised from 4.57 ± 0.93 to 6.02 ± 0.93 nm. As was described above, the different crystallite sizes calculated using FWHM and peak position can be explained by the strain effect appearing in crystallites.

**Figure 4:**
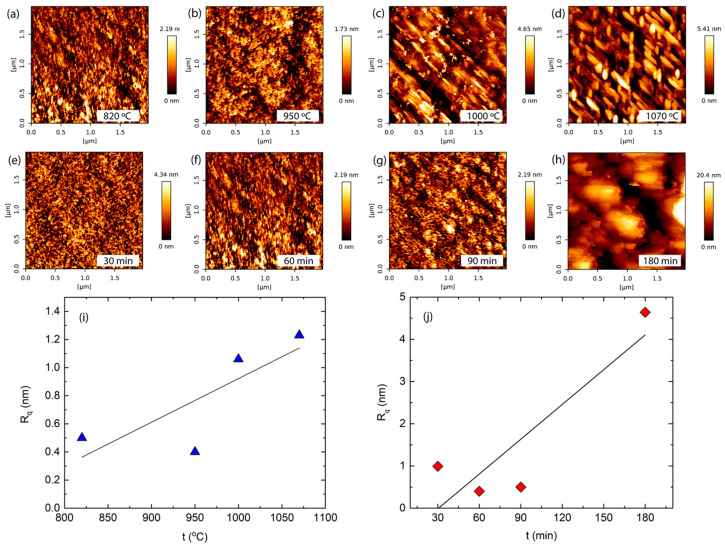
AFM images of samples deposited at (**a**) 820 °C, (**b**) 950 °C, (**c**) 1000 °C, and (**d**) 1070 °C temperatures and at a constant deposition time of 60 min. AFM images of samples deposited at (**e**) 30 min, (**f**) 60 min, (**g**) 90 min, and (**h**) 180 min times and at a constant deposition temperature of 820 °C. The surface roughness dependence on deposition temperature and time (**i**,**j**).

Figure 4 shows AFM pictures, where (a–d) are from samples deposited at different temperatures (at a constant deposition time of 60 min) and (e–h) are from samples deposited at different times (from 30 to 180) when deposition temperature was constant at 820 °C. Meanwhile, Figure 4i,j shows surface roughness Rq dependent on deposition temperature and time. In (a–d), we see that deposition temperature influences the size of grains. So, at 820 °C, we see a fine-grained structure. It was determined by analyzing AFM images that there are 1–2 nm high and 20–40 nm wide elements and their derivatives (Figure 4a). At 950 °C temperature, pits and grains are seen. The pits’ depth is 0.5 nm and the width is 25–30 nm. The height of the grains is 0.5–1 nm and the width is 25–30 nm (Figure 4b). A deposition temperature increase to 1000 °C results in 2–3.5 nm height and 130 nm width ribbons consisting of 200 nm long segments (Figure 4c). At 1070 °C temperature, we can observe interlaced grains of 50 nm width, 150 nm length, and 4–6 nm height (Figure 4d). In Figure 4e–h, we see that deposition time influences the size of grains. A fine-grained structure was grown after 30 min of deposition (at a constant 820 °C temperature). Grain height is up to 2 nm and width is 15–20 nm (Figure 4e). After 60 min deposition, 0.5 nm depth and 25–30 nm width pits and 0.5–1 nm height and 25–30 nm width grains are seen (Figure 4f). After 90 min, growth elements and their derivatives of 1–2 nm height and 20–40 nm width dominate (Figure 4g). The AFM image drastically changed after 180 min deposition—structural elements of 15–20 nm height and 320–360 nm width are seen (Figure 4h). Although the increase in grain size during the increase in time contradicts measurements of crystallites using Raman spectroscopy, it can be explained that grains are conglomerates that consist of nanocrystallites. That can be seen in Figure 4h, where grains consist of smaller objects corresponding to sizes determined by Raman spectroscopy. Figure 4i shows surface roughness Rq dependent on deposition temperature (at a constant deposition time of 60 min). Rq increases (from 0.5 to 1.25 nm) with increasing temperature. The effect of deposition time is similar—apart from fluctuations at 30–90 min, we see a strong roughness increase from ~0.5 to 4.7 nm (Figure 4j).

**Figure 5:**
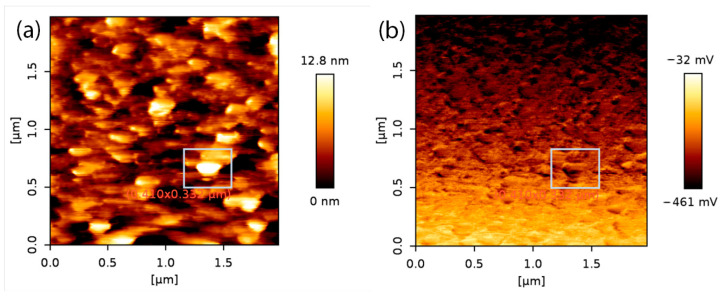
AFM (**a**) and LFM (**b**) images of sample deposited at 820 °C temperature, 60 min time, and 197 sccm nitrogen gas flow.

In Figure 5, AFM (a) and lateral force microscopy (LFM) (b) images of the sample deposited at 820 °C temperature, 60 min growth time, and 197 sccm nitrogen gas flow are shown. We see structural elements of 10–15 nm height, 180–200 nm width, and 200–240 nm length. Comparing the image of a sample deposited at the same conditions (Figure 4a) but with a different gas flow (152 sccm), we see that an increase in nitrogen gas flow strongly influences the grain size. The image is similar to Figure 4h; only lateral force microscopy shows (at the right) that the surface has visible hexagonal structures (marked areas). That is typical of a pure boron nitride surface [20]. 

**Figure 6:**
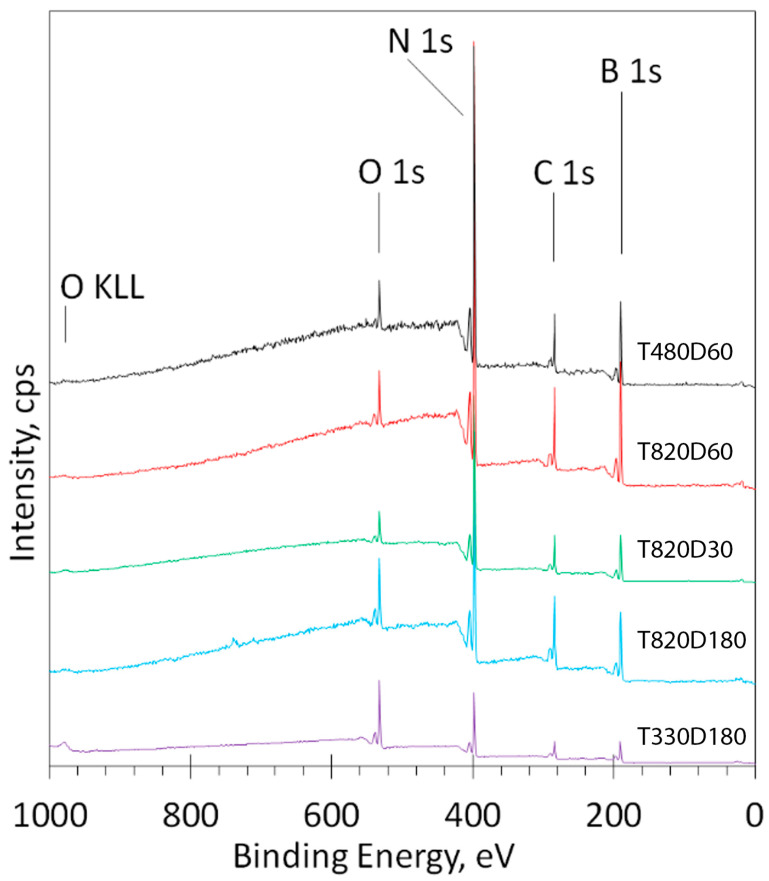
Comparison of the XPS survey spectra for several (as examples) samples; the elements and sample numbers are indicated for each curve.

For the surface chemical composition evaluation, the samples were analyzed using XPS. The survey spectra for all samples were collected and compared. In Figure 6, spectra for several samples are depicted. The spectra showed very similar patterns for all samples; only the intensity of the main peaks for nitrogen and boron was different, according to the calculated surface atomic concentrations (Table 1).

**Figure 7:**
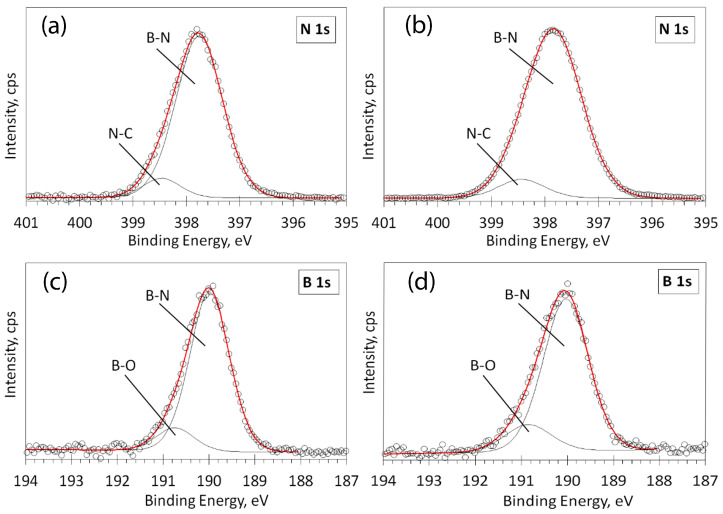
Deconvolution of high-resolution XPS spectra in the N 1s and B 1s regions: for the sample T820D60, deposited at 820 °C temperature, 60 min time, and 152 sccm nitrogen gas flow (**a**,**c**); for the sample T820D60N, deposited at 820 °C temperature, 60 min time, and 197 sccm nitrogen gas flow (**b**,**d**). Circles—acquired spectra; red line—envelope; thin black lines—fitted peaks.

High-resolution XPS spectra in the N 1s and B 1s regions were scanned and deconvoluted for chemical bond detection (Figure 7). Figure 7a shows the spectra of the sample T820D60, deposited at 820 °C temperature, 60 min time, and 152 sccm nitrogen gas flow. Figure 7b shows the spectra of the sample T820D60N, deposited at the same conditions but with a larger nitrogen gas flow (197 sccm). In Figure 7a,b, the main peak at 397.8 eV indicates that most of the nitrogen is bonded to boron, as described in the literature [51,52,53]. The low-intensity peak at 398.5 eV was attributed to N-C bonds [51,52,53].

Figure 7c,d presents the deconvolution of high-resolution XPS spectra in the B 1s region for the same T820D60 and T820D60N samples. The main peak at 190 eV indicates that most of the boron is bonded to nitrogen, in agreement with nitrogen bonds in the N 1s region. The position of this peak corresponds to known values of B-N bonds reported in the literature [51,52,53]. The low-intensity peak at 190.8 eV could be attributed to B-O bonds [51,52] due to adsorbed atmospheric oxygen. 

## 3. Materials and Methods

The boron nitride thin films were synthesized by the reactive high-power impulse magnetron sputtering (Hippies) method. The initial vacuum pressure was 8 × 10^−6^ mBar. After reaching the initial vacuum, nitrogen (or nitrogen mixture with argon) gas was injected into the vacuum chamber. A too low or too high working pressure does not allow ignition of plasma. The working pressure was 9 × 10^−3^ and 1.8 × 10^−2^ mBar. It should be mentioned that, under normal conditions (room temperature), igniting the plasma was impossible due to the high resistivity of the boron cathode. Therefore, typically, the boron cathode is sputtered using RF magnetron sputtering systems [54,55,56] or impurities-added boron LaB_6_ [32] and B_4_C [57] cathodes are used. In our case, for ignition of plasma and carrying out the sputtering process, the boron cathode was heated with a heat lamp (at an angle of 45° and a distance of 20 cm from the cathode) in a vacuum before starting the process. The boron cathode was isolated from the cooling of the magnetron using thin (0.5 mm) quartz plates. After heating the boron cathode to a temperature of 100 °C, the resistivity of boron decreases up to 30 times (as was measured before). As a result, the plasma ignites and the plasma discharge maintains the elevated temperature of the cathode. An unbalanced magnetron (Milko Angelov Consulting Co., Plovdiv, Bulgaria) with a high-purity (99.99%) boron target (Kurt J. Lesker Company GmbH, Dresden, Germany) was used. The pulse DC power controller SPIK2000A (Melec GmbH, Baden-Baden, Germany) was applied to generate high-power pulses. Prime-grade double-sided polished n-type monocrystalline Si (100) wafers (Sil’tronix Silicon Technologies, Archamps, France) were used as a substrate. The substrate was placed parallel to the plane of the cathode at a distance of 15 cm. Impulse parameters were chosen: t_On_ = 17 μs, t_Of_ = 150 μs, impulse current I = 1.2 A. The average current was constant during all processes, about ~0.12 A, and the average voltage was ~930 V. The pulse parameters were chosen as such because obtaining the boron nitride phase was impossible when the t_off_-to-t_on_ ratio was too low. The deposition time was chosen from 30 to 180 min (the shorter time gives too thin a film for Raman measurements, and growth time over 180 min results in no apparent changes in structure). The h-BN thin films’ synthesis requires an appropriate temperature, and it is in a relatively wide range (500~1000 °C) [58,59,60,61], depending on the method and other parameters. Our purpose was to investigate the broadest possible range of temperatures for the case of our method. Samples were deposited on substrates at different temperatures (200 to 1050 °C) using different deposition times. Detailed deposition conditions are listed in Table 2. The film thickness was determined using a laser ellipsometer Gaertner L-115 operating with a He–Ne laser (λ = 632.8 nm). Raman scattering measurements were performed using a Raman microscope inVia (Renishaw Wotton-under-Edge, UK). The excitation beam from a diode laser of 532 nm wavelength was focused on the sample using a 50 × objective (NA = 0.75, Leica, Solms, Germany). Laser power at the sample surface was 1.75 mW, integration time was 10 s or 100 s, and the signal was accumulated once. The Raman Stokes signal was dispersed with a diffraction grating (2400 grooves/mm), and data were recorded using a Peltier—cooled charge-coupled device (CCD) detector (1024 × 256 pixels). The Raman setup in both Raman wavenumber and spectral intensity was calibrated using silicon. We used the Levenberg–Marquardt method to calculate the best-fit parameters that minimize the weighted mean square error between the observations in Y and the best nonlinear fit. The two main parameters of the Gauss function, full width at half maximum (FWHM) and peak center, were calculated using this method.

From the fitted Raman spectra, using two parameters (FHWM and center position (shifting of central position from largest values of crystallites—Δ)), the crystallite size L_a_ of the hBN films can be estimated by extending the Nemanich model for hBN microcrystallites to hBN films [41]. This method also was reported in a few other studies [62,63,64]. According to the Nemanich model for hBN microcrystallites [41] from the FWHM and Δ values:

(1) $$L_{a}=\frac{1417}{\mathrm{FWHM}-8.70}$$

(2) $$L_{a}=\frac{380\cdot 10^{-8}}{∆+0.29}$$

Raman scattering measurements were performed at least 3 times in different sample places, and the average values were calculated. The luminescence hump and luminescence background of the Raman scattering spectra of different h-BN films were estimated by calculating the ratio of the luminescence hump maximum intensity and h-BN Raman peak intensity, as well as the ratio of the luminescence background line slope and h-BN Raman peak intensity. Atomic force microscopy (AFM) experiments were carried out at room temperature using a NanoWizardIII atomic force microscope (JPK Instruments, Bruker Nano GmbH, Berlin, Germany). At the same time, the data were analyzed using JPKSPM Data Processing software (Version spm-4.3.13, JPK Instruments, Bruker Nano GmbH). The AFM images were collected using an ACTA (Applied NanoStructures, Inc., Mountain View, CA, USA) probe (silicon cantilever shape—pyramidal, the radius of curvature (ROC) < 10.0 nm and cone angle 20°; reflex side coating—Al with a thickness of 50 ± 5 nm, force constant ~40 N m^−1^, and resonance frequency in the range of 300 kHz). Height, amplitude, and lateral imaging were recorded using steps with scan sizes of 2 μm and scan speeds of 1 Hz. The integral gain was set as 2, while the proportional gain was set as 5. Pixels for samples and lines were 516 × 516, operating in contact mode. The film’s surface composition was analyzed using the X-ray photoelectron spectroscopy (XPS) method. An X-ray photoelectron spectrometer XSAM800 (Kratos, Manchester, UK) equipped with a nonmonochromatic Al Ka radiation (1486.6 eV) excitation source was used for surface atomic calculations and survey spectra. A hemispherical electron energy analyzer was set to fixed analyzer transition (FAT) mode and 20 eV pass energy. A 0.5 eV increment of binding energy was used to acquire the survey. The energy scale of the system was calibrated using the peak positions of Au 4f7/2, Ag 3d5/2, and Cu 2p3/2. The base pressure in the analytical chamber was less than 5.8 × 10^−8^ Pa. Thermo Scientific ESCALAB 250Xi spectrometer with monochromatic Al Kα radiation (hν = 1486.6 eV) excitation was used for high-resolution spectra measurements and curve-fitting procedure. The hemispherical electron energy analyzer pass energy value of 20 eV was used. The energy scale of the system was calibrated with respect to Au 4f7/2, Ag 3d5/2, and Cu 2p3/2 peak positions. ESCALAB 250Xi Avantage software V5 was used for the peak deconvolution. All spectra fitting procedures were performed using symmetrical peaks and a 70:30 Gauss–Lorentz function ratio, except for the graphitic carbon peak, which was fitted using an asymmetrical peak shape and a Lorentzian–Gaussian function at a 70:30 ratio.

## 4. Discussion and Conclusions

As was mentioned above, a significant luminescence hump and luminescence background was seen in the Raman spectra of most h-BN films studied in this research. In this case, h-BN films contained at least 15 at.% of carbon. It should be mentioned that, in the case of the h-BN films deposited by reactive magnetron sputtering, a significant amount of carbon or oxygen impurities were found in numerous studies. Notably, the carbon content in h-BN films grown by RF reactive magnetron sputtering was as high as 20.83 at.%, and it decreased to about 8.98 at.% after the surface cleaning by argon ion [65]. In [66], the total amount of oxygen and carbon in the magnetron-sputtering-deposited h-BN films was much higher than in our study, in the 31–69 at.% range. A significant amount of carbon impurity in h-BN films was reported in [67]. In [68], the B-O component in the B1s peak was stronger than in our case, and the C-N fitting component area of the N1s peak was similar to that observed in our study. Chng, S. S. et al. found a significant amount of oxygen in most magnetron-sputtering-deposited h-BN films investigated in their study [36]. Carbon atomic concentration in h-BN films was decreased below 5 at.% only after selecting the appropriate additional hydrogen gas flow [69]. Thus, in the case of magnetron-sputter-deposited h-BN films, deposition conditions must be optimized to avoid film contamination by carbon or oxygen. In our case, the carbon amount in the films was minimized after the increase in the nitrogen gas flow and the related significant increase in the work pressure. Thus, the effects of the residual gas, along with the possible presence of the carbon-containing adsorbates, can be supposed. 

**Figure 8:**
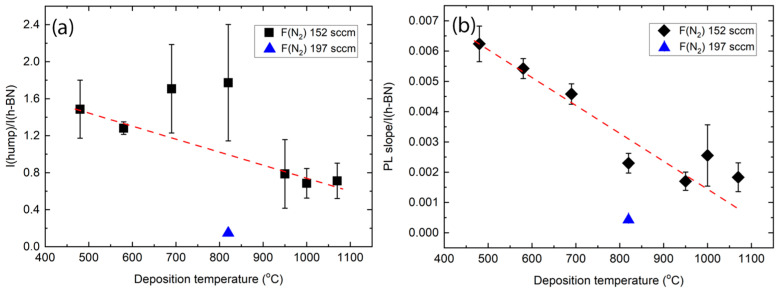
Luminescence hump maximum intensity and h-BN intensity ratio (**a**) and photoluminescence background line slope ratio with the h-BN peak intensity (**b**). Blue point refers to the h-BN film deposited using 197 sccm nitrogen gas flow.

Regarding the peculiarities of the Raman spectra of the h-BN films deposited in our study, it should be mentioned that the Raman spectra, very similar to those of the h-BN films grown in the present study at lower temperatures, were reported in [70]. Notably, Raman scattering spectra of h-BN films produced by vacuum annealing of the borazine amine polymer at 1600 °C temperature contained both h-BN-related sharp peak and a very broad luminescence hump with a maximum at ~2400 cm^−1^ [70]. It should be mentioned that a very broad Raman peak without any characteristic bands was observed for BC_x_N (0 < x < 2) films deposited by plasma-enhanced chemical vapor deposition in the 1000–3000 cm^−1^ range [61]. It was attributed to the fluorescence from the h-BN defects without indicating the nature of those defects. A luminescence hump or luminescence background was reported for the h-BN flake implanted by high-energy Ga ions and annealed at 820 °C [71]. It was explained by defect migration due to the annealing at 850 °C and the resulting transformation of the boron vacancies to the anti-site nitrogen vacancy complex (N_B_V_N_) defects [71]. However, in our case, the luminescence hump and background are more pronounced for films deposited at a temperature below 850 °C, contradicting the [71] hypothesis. A broad Raman peak with a maximum in the 1150–1400 cm^−1^ range was reported for amorphous BN films containing up to 15 at.% of carbon [72]. It should be noted that the B-H stretching modes can be found at 2291 cm^−1^ and 2382 cm^−1^, respectively [73]. Meanwhile, positions of the N-H bond vibration-related bands seem to be beyond the luminescence hump range reported in the present study (3176 cm^−1^, 3251 cm^−1^, and 3312 cm^−1^ wavenumbers) [73]. Thus, the luminescence hump can be partially related to the presence of the B-H bonds. On the other hand, in the present study, a luminescence hump was found for samples containing >15 at.% of carbon. At the same time, it was absent for samples containing less than 5 at.% of carbon. It is in good accordance with the studies mentioned above, in which a luminescence hump was reported for boron nitride films containing a significant amount of carbon [61,72]. The position of the C-C-bond-related Raman peaks is usually below 1700 cm^−1^ in amorphous carbon films [74]. However, C-H bond vibrations related to Raman peaks can be observed in the 2000–2200 cm^−1^ range [75]. The luminescence background can also be associated with the C-H bonds. Particularly, the ratio of the slope of the Raman spectra luminescence background line and G peak intensity is proportional to the bonded hydrogen amount in the diamond-like carbon films [74,76], and even a significant luminescence background slope with no characteristic Raman peaks was reported for hydrogenated amorphous carbon films containing >40 at.% of hydrogen [74,77]. The bonded hydrogen amount in the film should decrease with the increase in deposition temperature due to the desorption of hydrogen atoms caused by the B-H and C-H bond breakage [78,79,80,81,82]. That is in accordance with the present study, as seen in Figure 8. Thus, the observed luminescence hump can be explained by the formation of B-H and C-H bonds, and the presence of C-H bonds can explain the luminescence background. In this case, the increase in deposition temperature results in faster hydrogen desorption and a subsequent decrease in the h-BN luminescence-related features of the Raman film spectra. However, the increase in nitrogen gas flow was the most effective measure resulting in a significant decrease in the carbon amount in the film and a disappearance of the luminescence hump and luminescence background. The main factor can be supposed to be the increase in work pressure, while the base pressure remained the same, causing the residual gas to have a decreased influence on the growing film composition. Therefore, much fewer carbon and hydrogen atoms were incorporated into the films, and the number of C-H as well as B-H bonds was significantly decreased. 

In conclusion, hexagonal boron nitride films were deposited by high-power impulse reactive magnetron sputtering. Too low a nitrogen gas flow resulted in the formation of films containing a significant amount of carbon and the formation of C-H and B-H bonds. Increased synthesis temperature resulted in hydrogen desorption, less intensive hydrogen-bond-related luminescence features in Raman spectra of the films, and increased h-BN crystallite size. At the same time, in boron nitride samples grown at temperatures higher than 900 °C, tensile strain can be induced due to the thermal stress. The rise in nitrogen gas flow resulted in a significantly reduced carbon amount, the disappearance of the luminescence features in Raman scattering spectra of deposited films, and the appearance of h-BN-related features in the lateral force microscopy images of the boron nitride films. That was explained by decreased residual gas influence due to increased work pressure. Thus, h-BN film deposition temperature and nitrogen gas flow must be optimized to grow h-BN films containing fewer impurities and a more considerable amount of the h-BN phase. 

## Figures and Tables

**Table 1:** Calculated surface atomic concentrations. Samples are named T (temperature °C), D (deposition time min.), and N—increased (197 sccm) nitrogen flow.

Sample	O 1s	N 1s	C 1s	B 1s
T331D180	18.7	26.4	16.9	38.0
T820D180	7.9	32.1	21.7	38.3
T820D30	6.1	39.0	15.9	38.9
T820D60	4.3	37.1	15.3	43.4
T480D60	5.4	40.9	15.1	38.6
T820D60N	5.85	43.65	3.29	47.2

**Table 2:** Deposition conditions. Samples named T (temperature °C), D (deposition time min.), and N—increased (197 sccm) nitrogen flow.

Sample	N_2_ Gas Flow, sccm	Working Pressure, mmBar	Deposition Temperature, °C	Deposition Time, min	Thickness, nm
T330D180	152	9.3 × 10^−3^	330	180	255 ± 15
T820D180	152	9.3 × 10^−3^	820	180	210 ± 40
T820D30	152	9.4 × 10^−3^	820	30	60 ± 10
T820D90	152	9.4 × 10^−3^	820	90	190 ± 20
T1000D60	152	9.3 × 10^−3^	1000	60	75 ± 5
T1070D60	152	9.4 × 10^−3^	1070	60	80 ± 20
T950D60	152	9.3 × 10^−3^	950	60	77 ± 7
T820D60	152	9.3 × 10^−3^	820	60	117 ± 3
T690D60	152	9.2 × 10^−3^	690	60	150 ± 10
T580D60	152	9.2 × 10^−3^	580	60	165 ± 15
T480D60	152	9.4 × 10^−3^	480	60	152 ± 12
T820D60N	197	1.8 × 10^−2^	820	60	107 ± 7

## Data Availability

The original contributions presented in this study are included in the article; further inquiries can be directed to the corresponding authors.

## References

[1] Taniguchi T., Sato T., Utsumi W., Kikegawa T., Shimomura O. (1997). In-situ X-ray Observation of Phase Transformation of Rhombohedral Boron Nitride under Static High Pressure and High Temperature. Diam. Relat. Mater..

[2] Golberg D., Bando Y., Huang Y., Terao T., Mitome M., Tang C., Zhi C. (2010). Boron Nitride Nanotubes and Nanosheets. ACS Nano.

[3] Hong S., Lee C.-S., Lee M.-H., Lee Y., Ma K.Y., Kim G., Yoon S.I., Ihm K., Kim K.-J., Shin T.J. (2020). Ultralow-Dielectric-Constant Amorphous Boron Nitride. Nature.

[4] Cui Z., He Y., Tian H., Khanaki A., Xu L., Shi W., Liu J. (2020). Study of Direct Tunneling and Dielectric Breakdown in Molecular Beam Epitaxial Hexagonal Boron Nitride Monolayers Using Metal–Insulator–Metal Devices. ACS Appl. Electron. Mater..

[5] Cai Q., Scullion D., Gan W., Falin A., Cizek P., Liu S., Edgar J.H., Liu R., Cowie B.C.C., Santos E.J.G. (2020). Outstanding Thermal Conductivity of Single Atomic Layer Isotope-Modified Boron Nitride. Phys. Rev. Lett..

[6] Kostoglou N., Polychronopoulou K., Rebholz C. (2015). Thermal and Chemical Stability of Hexagonal Boron Nitride (h-BN) Nanoplatelets. Vacuum.

[7] Ares P., Cea T., Holwill M., Wang Y.B., Roldán R., Guinea F., Andreeva D.V., Fumagalli L., Novoselov K.S., Woods C.R. (2020). Piezoelectricity in Monolayer Hexagonal Boron Nitride. Adv. Mater..

[8] Falin A., Cai Q., Santos E.J.G., Scullion D., Qian D., Zhang R., Yang Z., Huang S., Watanabe K., Taniguchi T. (2017). Mechanical Properties of Atomically Thin Boron Nitride and The Role of Interlayer Interactions. Nat. Commun..

[9] Weng Q., Wang X., Wang X., Bando Y., Golberg D. (2016). Functionalized hexagonal boron nitride nanomaterials: Emerging properties and applications. Chem. Soc. Rev..

[10] Britnell L., Gorbachev R.V., Jalil R., Belle B.D., Schedin F., Mishchenko A., Georgiou T., Katsnelson M.I., Eaves L., Morozov S.V. (2012). Field-Effect Tunneling Transistor Based on Vertical Graphene Heterostructures. Science.

[11] Gao T., Song X., Du H., Nie Y., Chen Y., Ji Q., Sun J., Yang Y., Zhang Y., Liu Z. (2015). Temperature-Triggered Chemical Switching Growth of In-Plane and Vertically Stacked Graphene-Boron Nitride Heterostructures. Nat. Commun..

[12] Han Z., Li M., Li L., Jiao F., Wei Z., Geng D., Hu W. (2021). When Graphene Meets White Graphene—Recent Advances in the Construction of Graphene and h-BN Heterostructures. Nanoscale.

[13] Watanabe K., Taniguchi T., Kanda H. (2004). Direct-Bandgap Properties and Evidence for Ultraviolet Lasing of Hexagonal Boron Nitride Single Crystal. Nat. Mater..

[14] Li L.H., Cervenka J., Watanabe K., Taniguchi T., Chen Y. (2014). Strong Oxidation Resistance of Atomically Thin Boron Nitride Nanosheets. ACS Nano.

[15] Piquemal-Banci M., Galceran R., Godel F., Caneva S., Martin M.-B., Weatherup R.S., Kidambi P.R., Bouzehouane K., Xavier S., Anane A. (2018). Insulator-to-Metallic Spin-Filtering in 2D-Magnetic Tunnel Junctions Based on Hexagonal Boron Nitride. ACS Nano.

[16] Ma K.Y., Kim M., Shin H.S. (2022). Large-Area Hexagonal Boron Nitride Layers by Chemical Vapor Deposition: Growth and Applications for Substrates, Encapsulation, and Membranes. Acc. Mater. Res..

[17] Liu H., You C.Y., Li J., Galligan P.R., You J., Liu Z., Cai Y., Luo Z. (2021). Synthesis of Hexagonal Boron Nitrides by Chemical Vapor Deposition and Their Use as Single Photon Emitters. Nano Mater. Sci..

[18] Fukamachi S., Solís-Fernández P., Kawahara K., Tanaka D., Otake T., Lin Y.-C., Suenaga K., Ago H. (2023). Large-Area Synthesis and Transfer of Multilayer Hexagonal Boron Nitride for Enhanced Graphene Device Arrays. Nat. Electron..

[19] Dąbrowska A.K., Binder J., Prozheev I., Tuomisto F., Iwański J., Tokarczyk M., Korona K.P., Kowalski G., Stępniewski R., Wysmołek A. (2024). Defects in Layered Boron Nitride Grown by Metal Organic Vapor Phase Epitaxy: Luminescence and Positron Annihilation Studies. J. Lumin..

[20] Yang X., Nitta S., Nagamatsu K., Bae S.-Y., Lee H.-J., Liu Y., Pristovsek M., Honda Y., Amano H. (2018). Growth of Hexagonal Boron Nitride on Sapphire Substrate by Pulsed-Mode Metalorganic Vapor Phase Epitaxy. J. Cryst. Growth.

[21] Li X., Sundaram S., El Gmili Y., Ayari T., Puybaret R., Patriarche G., Voss P.L., Salvestrini J.P., Ougazzaden A. (2016). Large-Area Two-Dimensional Layered Hexagonal Boron Nitride Grown on Sapphire by Metalorganic Vapor Phase Epitaxy. Cryst. Growth Des..

[22] Cheng T.S., Summerfield A., Mellor C.J., Khlobystov A.N., Eaves L., Foxon C.T., Beton P.H., Novikov S.V. (2018). High-Temperature Molecular Beam Epitaxy of Hexagonal Boron Nitride with High Active Nitrogen Fluxes. Materials.

[23] Vuong T.Q.P., Cassabois G., Valvin P., Rousseau E., Summerfield A., Mellor C.J., Cho Y., Cheng T.S., Albar J.D., Eaves L. (2017). Deep Ultraviolet Emission in Hexagonal Boron Nitride Grown by High-Temperature Molecular Beam Epitaxy. 2D Mater..

[24] Rigato V., Spolaore M., Della Mea G. (2011). Deposition of Boron Nitride Coatings by Reactive Rf Magnetron Sputtering: Correlation Between Boron and Nitrogen Contents and the Flux of Energetic Ar+ Ions at the Substrate. MRS Proc..

[25] Oks E., Anders A., Nikolaev A., Yushkov Y. (2017). Sputtering of Pure Boron Using a Magnetron Without a Radio-Frequency Supply. Rev. Sci. Instrum..

[26] Vetter J., Shimizu T., Kurapov D., Sasaki T., Mueller J., Stangier D., Esselbach M. (2023). Industrial Application Potential of High Power Impulse Magnetron Sputtering for Wear and Corrosion Protection Coatings. J. Appl. Phys..

[27] Olejníček J., Šmíd J., Perekrestov R., Kšírová P., Rathouský J., Kohout M., Dvořáková M., Kment Š., Jurek K., Čada M. (2019). Co_3_O_4_ Thin Films Prepared by Hollow Cathode Discharge. Surf. Coat. Technol..

[28] Kipkirui N.G., Lin T.-T., Kiplangat R.S., Lee J.-W., Chen S.-H. (2022). HiPIMS and RF magnetron sputtered Al_0.5_CCrFeNi_2_Ti_0.5_ HEA Thin-Film Coatings: Synthesis and Characterization. Surf. Coat. Technol..

[29] Hossain M.D., Borman T., Mcllwaine N.S., Maria J.-P. (2022). Bipolar High-Power Impulse Magnetron Sputtering Synthesis of High-entropy carbides. J. Am. Ceram. Soc..

[30] Loquai S., Baloukas B., Klemberg-Sapieha J.E., Martinu L. (2017). HiPIMS-Deposited Thermochromic VO_2_ Films with High Environmental Stability. Sol. Energy Mater. Sol. Cells.

[31] Whiteside M., Arulkumaran S., Chng S.S., Shakerzadeh M., Teo H.T.E., Ng G.I. (2020). On the Recovery of 2DEG Properties in Vertically Ordered h-BN Deposited AlGaN/GaN Heterostructures on Si Substrate. Appl. Phys. Express.

[32] Cometto O., Sun B., Tsang S.H., Huang X., Koh Y.K., Teo E.H.T. (2015). Vertically Self-Ordered Orientation of Nanocrystalline Hexagonal Boron Nitride Thin Films for Enhanced Thermal Characteristics. Nanoscale.

[33] Chng S.S., Zhu M., Du Z., Wang X., Whiteside M., Ng Z.K., Shakerzadeh M., Tsang S.H., Teo E.H.T. (2020). Dielectric Dispersion and Superior Thermal Characteristics in Isotope-Enriched Hexagonal Boron Nitride Thin Films: Evaluation as Thermally Self-Dissipating Dielectrics for GaN Transistors. J. Mater. Chem. C.

[34] Whiteside M., Arulkumaran S., Ng G.I. (2021). Demonstration of Vertically-Ordered h-BN/AlGaN/GaN Metal-Insulator-Semiconductor High-Electron-Mobility Transistors on Si Substrate. Mater. Sci. Eng. B.

[35] Zhang H., Ju X., Jiang H., Yang D., Wei R., Hu W., Lu X., Zhu M. (2024). Implementation of High Thermal Conductivity and Synaptic Metaplasticity in Vertically-Aligned Hexagonal Boron Nitride-Based Memristor. Sci. China Mater..

[36] Chng S.S., Zhu M., Wu J., Wang X., Ng Z.K., Zhang K., Liu C., Shakerzadeh M., Tsang S., Teo E.H.T. (2020). Nitrogen-Mediated Aligned Growth of Hexagonal BN Films for Reliable High-Performance InSe Transistors. J. Mater. Chem. C.

[37] Hahn J., Friedrich M., Pintaske R., Schaller M., Kahl N., Zahn D.R.T., Richter F. (1996). Cubic Boron Nitride Films by d.c. and r.f. Magnetron Sputtering: Layer Characterization and Process Diagnostics. Diam. Relat. Mater..

[38] Androulidakis C., Koukaras E.N., Poss M., Papagelis K., Galiotis C., Tawfick S. (2018). Strained Hexagonal Boron Nitride: Phonon shift and Gr\”uneisen parameter. Phys. Rev. B.

[39] Li L.H., Chen Y. (2016). Atomically Thin Boron Nitride: Unique Properties and Applications. Adv. Funct. Mater..

[40] Chen X., Luan K., Zhang W., Liu X., Zhao J., Hou L., Gao Y., Song J., Chen Z. (2021). Effect of Employing Chromium as a Buffer Layer on the Crystallinity of Hexagonal Boron Nitride Films Grown by LPCVD. J. Mater. Sci. Mater. Electron..

[41] Nemanich R.J., Solin S.A., Martin R.M. (1981). Light Scattering Study of Boron Nitride Microcrystals. Phys. Rev. B.

[42] Chen X., Sun H., Zhang W., Tan C., Liu X., Zhao J., Hou L., Gao Y., Song J., Chen Z. (2022). The effects of Post-Annealing Technology on Crystalline Quality and Properties of Hexagonal Boron Nitride Films Deposited on Sapphire Substrates. Vacuum.

[43] Chen X., Tan C., Liu X., Luan K., Guan Y., Liu X., Zhao J., Hou L., Gao Y., Chen Z. (2021). Growth of Hexagonal Boron Nitride Films on Silicon Substrates by Low-Pressure Chemical Vapor Deposition. J. Mater. Sci. Mater. Electron..

[44] Zhou H., Zhu J., Liu Z., Yan Z., Fan X., Lin J., Wang G., Yan Q., Yu T., Ajayan P. (2014). High Thermal Conductivity of Suspended Few-Layer Hexagonal Boron nitride Sheets. Nano Res..

[45] Wang W., Li Z., Marsden A.J., Bissett M.A., Young R.J. (2021). Interlayer and Interfacial Stress Transfer in hBN Nanosheets. 2D Mater..

[46] Becton M., Wang X. (2015). Grain-Size Dependence of Mechanical Properties in Polycrystalline Boron-Nitride: A Computational Study. Phys. Chem. Chem. Phys..

[47] Paul R., Tasnim T., Dhar R., Mojumder S., Saha S., Motalab M.A. Study of Uniaxial Tensile Properties of Hexagonal Boron Nitride Nanoribbons. Proceedings of the TENCON 2017–2017 IEEE Region 10 Conference.

[48] Bera K., Chugh D., Patra A., Tan H.H., Jagadish C., Roy A. (2020). Strain Distribution in Wrinkled hBN Films. Solid State Commun..

[49] Duan X., Yang Z., Chen L., Tian Z., Cai D., Wang Y., Jia D., Zhou Y. (2016). Review on the Properties of Hexagonal Boron Nitride Matrix Composite Ceramics. J. Eur. Ceram. Soc..

[50] Zhang X., Yue J., Chen G., Yan H. (1998). Study on Stress and Strain of Cubic Boron Nitride Thin Films. Thin Solid Films.

[51] Chen M., Zhang Q., Fang C., Shen Z., Lu Y., Liu T., Tan S., Zhang J. (2023). Influence of Sapphire Substrate with Miscut Angles on Hexagonal Boron Nitride Films Grown by Halide Vapor Phase Epitaxy. CrystEngComm.

[52] Sharma K.P., Sharma S., Khaniya Sharma A., Paudel Jaisi B., Kalita G., Tanemura M. (2018). Edge Controlled Growth of Hexagonal Boron Nitride Crystals on Copper Foil by Atmospheric Pressure Chemical Vapor Deposition. CrystEngComm.

[53] Naumkin A.V., Kraut-Vass A., Powell C.J., Gaarenstroom S.W., National Institute of Standards and Technology (2012). NIST X-ray Photoelectron Spectroscopy Database, Version 4.1..

[54] Deng J., Wang B., Tan L., Yan H., Chen G. (2000). The Growth of Cubic Boron Nitride Films by RF Reactive Sputter. Thin Solid Films.

[55] Singh M., Vasudev H., Kumar R. (2020). Microstructural Characterization of BN Thin Films Using RF Magnetron Sputtering Method. Mater. Today Proc..

[56] Mieno M., Yoshida T. (1990). Preparation of Cubic Boron Nitride Films by RF Sputtering. Jpn. J. Appl. Phys..

[57] Schütze A., Bewilogua K., Lüthje H., Kouptsidis S., Gaertner M. (1997). Improvement of the Adhesion of Sputtered Cubic Boron Nitride Films. Surf. Coat. Technol..

[58] Liu D., Chen X., Yan Y., Zhang Z., Jin Z., Yi K., Zhang C., Zheng Y., Wang Y., Yang J. (2019). Conformal Hexagonal-Boron Nitride Dielectric Interface for Tungsten Diselenide Devices with Improved Mobility and Thermal Dissipation. Nat. Commun..

[59] Wei D., Peng L., Li M., Mao H., Niu T., Han C., Chen W., Wee A.T.S. (2015). Low Temperature Critical Growth of High Quality Nitrogen Doped Graphene on Dielectrics by Plasma-Enhanced Chemical Vapor Deposition. ACS Nano.

[60] Wei D., Lu Y., Han C., Niu T., Chen W., Wee A.T.S. (2013). Critical Crystal Growth of Graphene on Dielectric Substrates at Low Temperature for Electronic Devices. Angew. Chem..

[61] Yi K., Jin Z., Bu S., Wang D., Liu D., Huang Y., Dong Y., Yuan Q., Liu Y., Wee A.T.S. (2020). Catalyst-Free Growth of Two-Dimensional BCxN Materials on Dielectrics by Temperature-Dependent Plasma-Enhanced Chemical Vapor Deposition. ACS Appl. Mater. Interfaces.

[62] Snure M., Paduano Q., Hamilton M., Shoaf J., Mann J.M. (2014). Optical Characterization of Nanocrystalline Boron Nitride Thin Films Grown by Atomic Layer Deposition. Thin Solid Films.

[63] Ahmed K., Dahal R., Weltz A., Lu J.-Q., Danon Y., Bhat I.B. (2016). Growth of Hexagonal Boron Nitride on (111) Si for Deep UV Photonics and Thermal Neutron Detection. Appl. Phys. Lett..

[64] Singhal R., Echeverria E., McIlroy D.N., Singh R.N. (2021). Synthesis of Hexagonal Boron Nitride Films on Silicon and Sapphire Substrates by Low-Pressure Chemical Vapor Deposition. Thin Solid Films.

[65] Quan H., Wang X., Zhang L., Liu N., Feng S., Chen Z., Hou L., Wang Q., Liu X., Zhao J. (2017). Stability to Moisture of Hexagonal Boron Nitride Films Deposited on Silicon by RF Magnetron Sputtering. Thin Solid Films.

[66] Hirata Y., Yoshii K., Yoshizato M., Akasaka H., Ohtake N. (2023). Developing a Synthesis Process for Large-Scale h-BN Nanosheets Using Magnetron Sputtering and Heat Annealing. Adv. Eng. Mater..

[67] Kang Y., Chen L., Liu C., Tang X., Zhu X., Gao W., Yin H. (2022). Enhancement of n-type Conductivity of Hexagonal Boron Nitride Films by In-Situ Co-Doping of Silicon and Oxygen. J. Phys. Condens. Matter.

[68] Chen R., Li Q., Zhang Q., Wang M., Fang W., Zhang Z., Yun F., Wang T., Hao Y. (2023). Electronic Properties of Vertically Stacked h-BN/B1–xAlxN Heterojunction on Si(100). ACS Appl. Mater. Interfaces.

[69] BenMoussa B., D’Haen J., Borschel C., Barjon J., Soltani A., Mortet V., Ronning C., D’Olieslaeger M., Boyen H.G., Haenen K. (2012). Hexagonal Boron Nitride Nanowalls: Physical Vapour Deposition, 2D/3D Morphology and Spectroscopic Analysis. J. Phys. D Appl. Phys..

[70] Rye R.R., Tallant D.R., Borek T.T., Lindquist D.A., Paine R.T. (1991). Mechanistic Studies of the Conversion of Borazine Polymers to Boron Nitride. Chem. Mater..

[71] Venturi G., Chiodini S., Melchioni N., Janzen E., Edgar J.H., Ronning C., Ambrosio A. (2024). Selective Generation of Luminescent Defects in Hexagonal Boron Nitride. Laser Photonics Rev..

[72] Gago R., Jiménez I., Agulló-Rueda F., Albella J.M., Czigány Z., Hultman L. (2002). Transition from Amorphous Boron Carbide to Hexagonal Boron Carbon Nitride Thin Films Induced by Nitrogen Ion Assistance. J. Appl. Phys..

[73] Kupenko I., Dubrovinsky L., Dmitriev V., Dubrovinskaia N. (2012). In Situ Raman Spectroscopic Study of the Pressure Induced Structural Changes in Ammonia Borane. J. Chem. Phys..

[74] Ferrari A.C., Robertson J., Ferrari A.C., Robertson J. (2004). Raman Spectroscopy of Amorphous, Nanostructured, Diamond–Like Carbon, and Nanodiamond. Philos. Trans. R. Soc. Lond. Ser. A Math. Phys. Eng. Sci..

[75] Tabata H., Fujii M., Hayashi S., Doi T., Wakabayashi T. (2006). Raman and Surface-Enhanced Raman Scattering of a Series of Size-Separated Polyynes. Carbon.

[76] Casiraghi C., Piazza F., Ferrari A.C., Grambole D., Robertson J. (2005). Bonding in Hydrogenated Diamond-Like Carbon by Raman Spectroscopy. Diam. Relat. Mater..

[77] Casiraghi C., Ferrari A.C., Robertson J. (2005). Raman Spectroscopy of Hydrogenated Amorphous Carbons. Phys. Rev. B.

[78] Hoang D.-Q., Pobedinskas P., Nicley S.S., Turner S., Janssens S.D., Van Bael M.K., D’Haen J., Haenen K. (2016). Elucidation of the Growth Mechanism of Sputtered 2D Hexagonal Boron Nitride Nanowalls. Cryst. Growth Des..

[79] Akkerman Z.L., Kosinova M.L., Fainer N.I., Rumjantsev Y.M., Sysoeva N.P. (1995). Chemical Stability of Hydrogen-Containing Boron Nitride Films Obtained by Plasma Enhanced Chemical Vapour Deposition. Thin Solid Films.

[80] Bounouh Y., Thèye M.L., Dehbi-Alaoui A., Matthews A., Stoquert J.P. (1995). Influence of Annealing on the Hydrogen Bonding and the Microstructure of Diamondlike and Polymerlike Hydrogenated Amorphous Carbon Films. Phys. Rev. B.

[81] Bounouh Y., Zellama K., Zeinert A., Benlahsen M., Clin M., Thèye M.L. (1997). Modes of Hydrogen Incorporation in Hydrogenated Amorphous Carbon (a–C:H), Modifications with Annealing Temperature. J. Phys. III Fr..

[82] Wang W.J., Wang T.M., Chen B.L. (1996). Hydrogen Release from Diamondlike Carbon Films Due to Thermal Annealing in Vacuum. Nucl. Instrum. Methods Phys. Res. Sect. B Beam Interact. Mater. At..

